# Implementation of a risk mitigating COVID-adapted colorectal cancer pathway

**DOI:** 10.1136/bmjoq-2020-001135

**Published:** 2021-01-11

**Authors:** Janice Miller, Laura J Thomson, Lisa S P Stewart, Jenny Fleming, Malcolm G Dunlop, Farhat V N Din, Yasuko Maeda

**Affiliations:** 1 Department of Colorectal Surgery, Western General Hospital, University of Edinburgh, Edinburgh, UK; 2 Quality Improvement Team, Western General Hospital, University of Edinburgh, Edinburgh, UK

**Keywords:** surgery, patient-centred care, crisis management, clinical practice guidelines, governance

COVID-19 has brought about unprecedented challenges to healthcare systems forcing them to meet the sudden increase in demand of large numbers of critically ill patients. However, the long-term negative impact will be on patients without COVID due to lost opportunities to undergo standard diagnostic testing and treatment in a timely manner.^1^

We introduced a COVID-adapted colorectal cancer pathway at the outset of the pandemic in an attempt to mitigate the risks of delayed and missed cancer diagnoses. The COVID-adapted pathway design was based on appraisal of the current literature and used the available non-aerosol generating testing tools, namely, quantitative faecal immunochemistry testing (qFIT) and CT scanning with oral preparation (figure 1)^2–4^ and triaged patients based on their symptomatic risk (high-risk symptoms, including palpable abdominal mass, persistent change in bowel habit to looser stool not just simple constipation, repeated rectal bleeding without an obvious benign anal cause or blood mixed in with the stool, abdominal pain with weight loss with or without iron deficiency anaemia). The qFIT test is routinely used for screening and as a triage tool in low-risk populations, however it is not used as a rule out test in those with potential colorectal cancer due to its sensitivity. The threshold of 10 µg/g was not used for investigation as data from several health boards suggest that the positivity rate is ~23%. We, therefore, used the threshold of 80 µg/g as based on the Scottish bowel screening guidelines.

**Figure 1:**
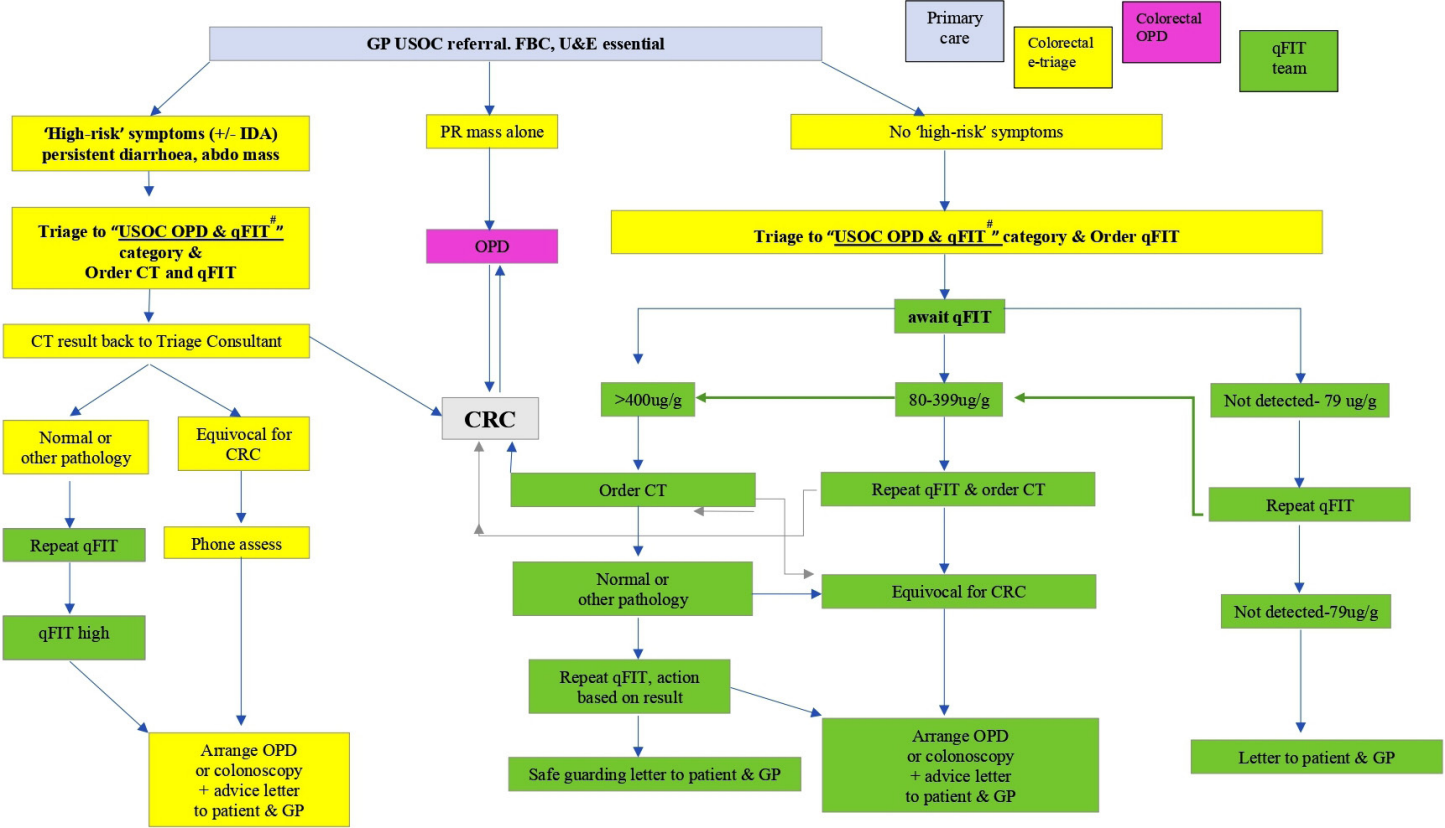
A summary of the COVID-adapted colorectal pathway. Patients were triaged by colorectal consultants with information provided from general practice. They proceeded through the pathway in a stepwise fashion being stratified by qFIT results. CRC, colorectal cancer; FBC, full blood count; GP, general practitionaer; IDA, iron deficiency anaemia; OPD, outpatients department; qFIT, quantitative faecal immunochemical test; USOC, urgent suspicion of cancer; U&E, urea and electrolytes.

## Pathway design and adoption

There were inevitable challenges to the design, implementation and operation of a new pathway during a time when the National Health Service (NHS) was put on to an emergency footing. While taking advantage of standard methodology and conceptual framework,^5^ some adjustments and improvisations were required to implement the pathway effectively. Swift agreement on broad principles and a clear single goal of mitigating risks for patients enabled powerful facilitation of the pathway. Due to the pressing need to implement this alternative pathway, wider consultation was not undertaken at the outset other than lead stakeholders. We were conscious that it could be difficult for clinicians to become familiar with the pathway design and be fluent at requesting the appropriate tests when they had not been directly involved in the design process. To circumvent this, an online manual was written, accompanied by a step-by-step procedural diagram.

## Pathway implementation

The main challenge of pathway implementation was the requirement for subsequent robust management. It required streamlining requests for tests, triaging patients who were referred urgently with symptoms suspicious of colorectal cancer and tracking patient flow. This was only possible with engagement from a wide multidisciplinary team, including consultant surgeons, gastroenterologists, radiologists and biochemists as well as general practitioners and specialist nursing staff and support from the NHS health board. A bespoke data management system was developed using Excel, which enabled us to have full grasp of activities and signpost patients in a stepwise manner to the tests required at each stage. It also allowed identification of those who did not return their qFIT within a specified time period or did not attend appointments. Staff members phoned these patients to clarify the rationale of the pathway and encourage uptake. Live data from the pathway allowed real-time analysis of pathway performance, including cancer detection rate, which was crucial from a clinical governance perspective.^6^ Demonstrating the benefit of the pathway regularly at departmental meetings helped clinical staff to engage with the process and retain responsibility for their patients.

**Figure 2:**
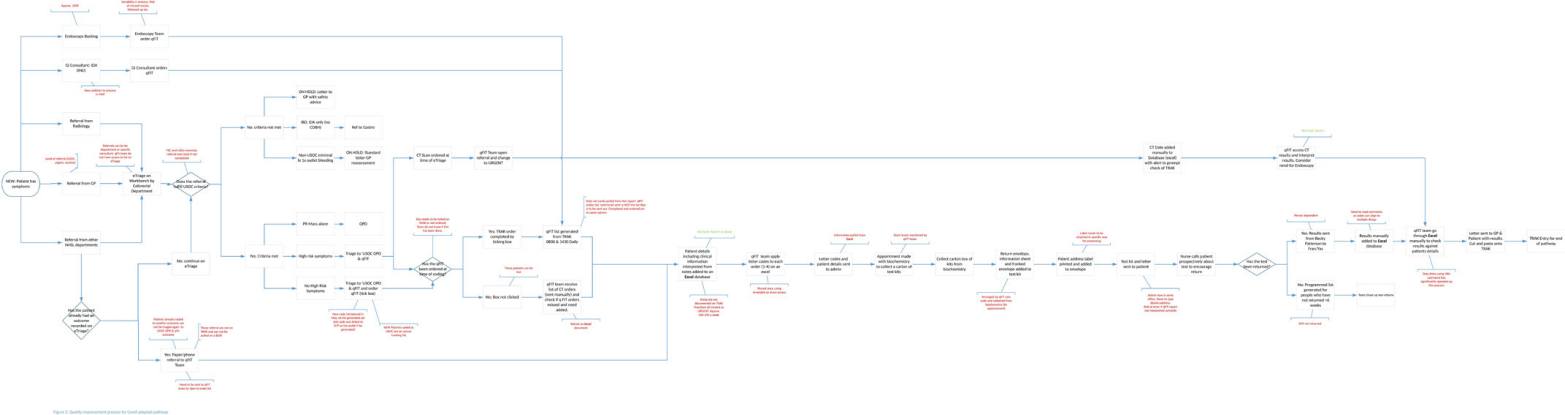
Mapping of the pathway process for quality improvement.

In parallel, the pathway operational steps were mapped out. Variation and wastage was identified by the quality improvement team using a quality management approach (figure 2). This provided a clear picture of where resources were required (eg, the level of staffing and infrastructure needed), and identified barriers and constraints requiring solutions. Regular feedback to management attempted to facilitate support and resource mobilisation. The pathway operation required flexibility as logistical and practical adjustments were required over time as some diagnostic services slowly resumed. Such fluidity and dynamism was achieved by a tightly knit operational team, including medical, nursing and administrative staff supported by effective leaders.

## Pathway results

At the outset, there were many unknown factors, including virulence or expected duration of the pandemic. These coupled with a lack of specific contextualised clinical data to make the pathway completely evidence based led to anxieties surrounding its performance. Patients were predominantly referred under the urgent suspicion of cancer (USOC) category (357), with 48 urgent and 17 routine referrals being upgraded to USOC by the triaging colorectal consultant. We detected cancers in 3.1% (13 cancers found in included 422 patients) of urgent referrals compared with the expected 3.2% from historical practice and significantly outperformed the situation should the pathway not have been in existence (0.18% cancer detection rate if the service was limited to emergencies only). It achieved the initial purpose and offered safety netting for more than 600 patients referred with alarm symptoms to a tertiary colorectal unit during the pandemic.

## Pathway maintenance long term

Maintenance of the pathway is likely to be required in the longer term given the enormity of the pandemic and the slow resumption of normal services. No patients were discharged on the basis of qFIT tests alone. All patients were safety netted either with CT scanning or they remained on the list for an outpatient appointment or colonoscopy. All patients received letters at each stage detailing their results and explaining any safety netting procedures they were to receive. This was done in conjunction with telephone consultations with specialist nurses. As previously shown implementation of new pathways is not straightforward and requires widespread agreement from many. Resource mobilisation, organisational adaptations and regular feedback were actively sought from an early stage and were essential to maintain change.^7^ This led to increased safety netting procedures being implemented (all patients proceeded to CT) when a variation in double qFIT testing was seen.

In conclusion, a transitional service change was required due to a rapid change in care provision for those patients referred with suspected colorectal cancer during the COVID-19 pandemic. Designing, implementing and maintaining a bespoke pathway was possible with strong leadership, adaptability of staff, flexible multidisciplinary team-working and wider support encouraged by regular feedback and review of performance.
